# Circulating apoE4 protein levels from dried blood spots predict cognitive function in a large population‐based survey setting

**DOI:** 10.1002/alz.14224

**Published:** 2024-09-05

**Authors:** Yacila I. Deza‐Lougovski, Luzia M. Weiss, Hannah M. Horton, Aijing Sun, Nis Borbye‐Lorenzen, Kristin Skogstrand, Solveig Holmgaard, Karen Andersen‐Ranberg, Vania Panes Lundmark, Axel Börsch‐Supan, Martina Börsch‐Supan, Anna Rieckmann

**Affiliations:** ^1^ Institute of Psychology, University of the Bundeswehr München Neubiberg Germany; ^2^ Max Planck Institute for Social Law and Social Policy Munich Germany; ^3^ Munich Research Institute for the Economics of Aging and SHARE Analyses (MEA) Munich Germany; ^4^ Department for Congenital Disorders Center for Neonatal Screening, Statens Serum Institut Copenhagen Denmark; ^5^ Department of Public Health Epidemiology, Biostatistics and Biodemography University of Southern Denmark Odense Denmark; ^6^ Department of Clinical Research Geriatric Research Unit University of Southern Denmark Odense Denmark; ^7^ Department of Integrative Medical Biology Umeå University Umeå Sweden; ^8^ Umeå Center for Functional Brain Imaging Umeå University Umeå Sweden; ^9^ Survey of Health, Ageing and Retirement in Europe (SHARE Biomarker Project) Munich Germany

**Keywords:** *APOE* ε4 allele, apoE4 protein, cognition, dried blood spots, SHARE

## Abstract

**INTRODUCTION:**

The *apolipoprotein E (APOE)* ε4 allele carries risk for cognitive impairment, but whether the level of circulating apoE4 protein in carriers affects cognition is unclear, as is how health and lifestyle impact circulating apoE4 levels.

**METHODS:**

We assayed apoE4 protein levels in dried blood spots of 12,532 adults aged 50+. Regression analyses tested the likelihood of cognitive impairment between groups and within those with detected apoE4 protein. Predictors of circulating apoE4 were assessed.

**RESULTS:**

We detected protein binding that indicates the presence of an *APOE* ε4 allele in 28.4% of this group. This group was more likely to have cognitive impairment, and this risk increases with age. However, higher apoE4 levels were associated with less likelihood of cognitive impairment within this group. Antihypertensive medication predicted apoE4 protein levels.

**DISCUSSION:**

The apoE4 isoform is associated with a deficient protein and worse cognition. This association is modulated by the level of circulating apoE4 protein in ε4 carriers.

**Highlights:**

- An assay to quantify apoE4 levels from dried blood spot samples was applied.
- The apoE4 protein was detected as specific binding at ≥30,000 pg/mL in 28.4% of samples.
- Having the apoE4 protein was associated with worse cognitive performance.
- Higher apoE4 protein levels in those who have it were associated with better cognition.
- Cardiovascular factors influenced levels of apoE4 protein.

## 1 BACKGROUND

The apolipoprotein E (apoE) is involved in a wide spectrum of biological functions. In the human body, the three major isoforms of apoE (apoE2, apoE3, apoE4) are encoded by the ε2, ε3, and ε4 alleles, respectively, on the long arm of chromosome 19.^1^ It is established that carriage of the *APOE* ε4 allele confers a higher risk for late‐onset Alzheimer's disease (AD) in a gene dose‐dependent manner.^2^ In addition, *APOE* ε4 influences cognition^3^ and differentially shapes brain function over the life span^4^, ^5^ even in the absence of neuropathological markers. Conversely, the *APOE* ε2 gene^6^ and specific variants of *APOE* ε3^7^, ^8^ confer a protective effect, perhaps by defending the brain from the harmful effects of lipid aggregation. Accumulating literature points to inefficient regulation of cellular homeostasis of lipids as one of the main mechanisms by which *APOE* ε4 confers age‐related vulnerability.^9^, ^10^ As such, carriage of the *APOE* ε4 allele is associated with a diminished capacity of the brain apoE protein to mediate the uptake of cellular cholesterol, potentially affecting amyloid‐β clearance and synaptic repair,^11^ though other mechanisms were proposed.^12^, ^13^ With an increased need to develop and monitor dementia biomarkers in routine analyses, where laboratory infrastructure is lacking,^14^ the current study focuses on the association between cognition and an apoE4 protein obtained from dried blood spots (DBS) samples in a survey setting.

The apoE protein is synthesized and secreted by several tissues. Outside the central nervous system (CNS), it is mainly produced by the liver, whereas astrocytes are the main source of apoE in the CNS.^15^ Even if peripheral and brain apoE are controlled by different pathways^16^ and peripheral apoE does not cross the blood‐brain barrier,^17^ prior research shows a correspondence between isoform‐specific concentrations of circulating and brain apoE proteins.^11^ Similarly, detection of circulating apoE4 in blood has been used as a proxy for the *APOE* ε4 genetic status.^18^, ^19^, ^20^

Gregg and colleagues demonstrated in human subjects that apoE4 is associated with a deficient lipoprotein metabolism.^21^ Later, animal models and work with cultured human astrocytoma suggested that the ratio of apoE4 to apoE3 in ε4/ ε3 carriers is comparable across plasma, brain, and cerebrospinal fluid samples. The apoE4 isoform makes up a smaller proportion (30%–40%) of total apoE, suggesting a preferential degradation of apoE4 compared with apoE3.^11^

In line, studies of human plasma samples showed that *APOE* ε4 carriers have significantly lower levels of plasma total apoE attributable to a specific decrease in the apoE4 isoform.^22^, ^23^, ^24^, ^25^ It remains unclear whether the level of blood apoE confers risk for cognitive impairment or AD. Whereas some studies reported significantly lower peripheral levels of apoE in AD patients (mostly *APOE* ε4 carriers) compared with controls,^26^ other studies showed that circulating apoE and apoE4 levels were neither correlated with cognition nor useful to distinguish AD patients from controls.^22^, ^27^ These inconsistencies could arise from the methodology used to detect or quantify apoE4 levels (e.g., immunoassays vs. mass spectrometry), the use of small samples, or a difference in metabolic pathways between peripheral and brain apoE.^16^ Similarly, findings relating circulating apoE4 to cardiovascular risk (i.e., another risk factor for dementia) are not conclusive, probably due to heterogeneous bindings between apoE and circulating lipids and lipoproteins.^28^

There are two novel contributions of this study: First, we report apoE4 from DBS samples and cognition data from a large population‐based survey. Samples were assayed to estimate apoE4 protein levels with a recently validated method, showing that only carriers of at least one copy of the *APOE ε4* allele have a specific binding to the apoE4 antibody at levels higher than 30,000 pg/mL (see^20^ and Supplementary S1; Methods). We hypothesized that individuals with specific binding would have more cognitive impairment than those without.^29^

Second, we explore the additional role of individual differences in levels of the apoE4 protein for cognition within the putative carrier group, as well as health‐ and lifestyle variables that modulate these differences.

## 2 METHODS

### 2.1 Study population

This study used cross‐sectional data from the Survey of Health, Ageing and Retirement in Europe—SHARE.^30^ SHARE is a large‐scale pan‐European survey (N∼140,000) that follows respondents aged 50 and older over time. The selected participants are a multi‐stage probability sample representative of the population 50 plus, drawn from the population registers that are available in the 28 SHARE countries. Once selected, the participation in the survey is voluntary upon informed consent. The response rate in the panel is about 85%. A person is excluded if she or he is incarcerated, hospitalized, or out of the country during the entire survey period, unable to speak the country's language(s), or has moved to an unknown address. For the survey, proxy interviews are used if the respondent is not able to do an interview, for example, for health reasons. Further detailed information about country‐specific ways to achieve full probability sampling can be found in Börsch‐Supan et al.^30^ For the present study, proxy respondents are excluded because an individual's performance on cognitive tests is the outcome of interest.

To provide objective health‐related measurements that complement the extensive self‐reported health data available in SHARE, a collection of DBS samples was implemented in Wave 6 in 2015.^31^ A total of 68,186 respondents (56.4% females) interviewed during this wave, 26,351 respondents from 12 countries (Israel, Spain, Greece, Italy, Switzerland, France, Slovenia, Belgium, Germany, Denmark, Estonia, Sweden) donated DBS samples. DBS were collected from all respondents who had previously participated in any SHARE wave and their partners. Participants who declined consent were unable to consent, or mentioned a medical reason to avoid blood collection were excluded from DBS. Further information is provided in Börsch‐Supan et al.^31^ Depending on the amount of blood on an individual DBS card, the blood sample could be analyzed for up to 17 different biomarkers (Biomarker type‐1: seven routine blood markers, type‐2: ten cyto‐ and neurokines, growth factors and apoE4). Analysis for apoE4 protein level and cognitive testing were available for a subsample of 12,532 respondents, 59.3% female and 40.7% male.

### 2.2 Definition of global cognitive impairment

Respondents performed a cognitive test battery that included verbal episodic memory (immediate and delayed recall), temporal orientation, verbal fluency (animals), and numeracy (serial subtractions). For a measure of global cognitive performance, test performance was Z‐scored in the entire cohort and then averaged across tests.^32^, ^33^ Cognitive impairment was defined by a cutoff of 1.5 standard deviations below the sample mean of the Z‐score of all tests. The categorical variable indicating global impairment (i.e., No = 0, Yes = 1) was used as the outcome in a multivariate logistic weighted model. In linear regression analyses and illustrations, the continuous variable for global impairment, as well as individual tests, were also considered as outcomes. A detailed description of the individual tests is provided in Supplementary S2; Methods.

**RESEARCH IN CONTEXT**

1. **Systematic review**: The authors conducted an extensive revision of the available human and animal literature on the apolipoprotein E4 (apoE4) protein isoform and its effects on cognition. The search was extended to findings describing the effect of all *APOE* alleles on total circulating apoE and specific circulating apoE isoforms in Alzheimer's disease (AD) patients and controls.
2. **Interpretation**: The amount of circulating apoE4 protein in ε4 carriers modulates the risk for cognitive decline. Specifically, high levels of the apoE4 protein protect from the risk associated with having the *APOE ε4* allele. Importantly, indicators of cardiovascular health modulate blood apoE4 protein level and might constitute a mechanism by which cardiovascular factors exacerbate dementia risk in ε4 carriers.
3. **Future directions**: Longitudinal approaches should be considered to elucidate whether cognitive trajectories in individuals with lower circulating apoE4 levels can be modified by interventions and healthy lifestyles. The use of weighted statistical models is important to account for sampling bias in cognitive aging.

### 2.3 DBS collection and analysis

The DBS method demonstrates numerous advantages over conventional, venous blood sampling in large international surveys collecting blood‐based biomarkers.^34^ A comprehensive description of the collection and country‐specific details can be found in Börsch‐Supan et al.^31^ Briefly, well‐trained interviewers prepared blood collection during the interview. After disinfection, the puncture site on a finger was pricked with a lancet. The first blood drop was discarded. The next blood drops were collected on respondent‐specific, barcode‐labeled filter cards (Ahlstrom 226). DBS were left to dry until the end of the SHARE interview, but at least 15 min before being packed with desiccant in plastic bags and mailed to the SHARE biobank (Odense, Denmark), where they were stored in freezers at −20°C until analyses.

The analyses of the apoE4 protein were carried out at the Statens Serum Institut in Copenhagen, Denmark. They are described in detail in Borbye‐Lorenzen et al.^20^ Briefly, DBS samples were extracted and assayed using a 10‐plex immunoassay using preprinted Meso‐Scale plates (Meso‐Scale Diagnostics [MSD], Maryland, USA) coated with antibodies specific for ApoE4 and nine other biomarkers reported elsewhere^35^ (see Supplementary S1; Methods). The apoE4 assay was based on an apoE4‐specific antibody for capture (#M067‐3, MBL International, Woburn, MA, USA) and an apoE‐pan‐specific antibody that detects apoE regardless of its isoform or genetic status (#MAB41441, R & D systems MSD) using recombinant Human apoE4 (#350‐04, Peprotech, lot# 316318—identical to #JM‐4699, MBL International) as calibrator. We incubated the preprinted MSD plates at room temperature (RT) with blocker A (#R93BA) for 30 min, and then washed them. The extracts were mixed 7:1 with 6× custom diluent (based on diluent 43, #R50AG, MSD) on the MSD plate. Calibrators were diluted in diluent 7 (#R54BB) and detection antibodies in diluent 3 (#R50AP, MSD). Controls were made in‐house from part of the calibrator solution in one batch, aliquoted in portions for each plate and stored at −20°C until use. We incubated the extracts on the MSD plate for 2 h at RT while shaking at 600 rpm before washing and the addition of detection antibody. This was followed by another 2 h of incubation at RT and 600 rpm. The samples were read on the QuickPlex SQ 120 (MSD) for 4 min after adding 2× Read buffer T (#R92TC, MSD). Analyte concentrations were calculated from the calibrator curves on each plate using 4PL logistic regression with the MSD Workbench software.

Previous work in a subsample (*N* = 554) of this study determined that a cutoff of 30,000 pg/mL apoE4 protein level distinguished specific versus non‐specific binding (i.e., from the genetic analysis it can be assumed with 97% sensitivity and 98% specificity that individuals with apoE4 protein levels ≥ 30,000 pg/mL carry at least one copy of the APOE ε4 allele whereas those with apoE4 protein levels <30,000 pg/mL do not and therefore the binding is unspecific).^20^ Applying this cutoff to the entire sample reported in this study, 3564 individuals showed specific binding.

Since the DBS samples were collected in non‐clinical environments, fieldwork conditions were recorded (i.e., outside temperature at the home of the respondent, drying time, shipment time, and humidity protection for the DBS sample^31^). Our prior work showed that temperature and blood spot size have a significant impact on apoE4 protein measures.^20^ Therefore, these variables were considered as covariates in the models that analyzed predictors of apoE4 levels.

### 2.4 Additional variables

Regression analyses considered several other covariates that are commonly associated with an increased vulnerability to cognitive decline and dementia in population‐based studies^36^, ^37^ and were available in SHARE. These included demographic variables (e.g., age, sex, years of education) and questions about lifestyle (social activities, physical activity), instrumental activities of daily living (IADLZA score^38^), a cardiovascular multimorbidity index as defined in Tai et al.,^39^ body mass index (BMI), depressive symptoms, self‐reported medical conditions (i.e., high cholesterol, hypertension, depression), and a list of current medications. The specific interview items and scoring are found in Supplementary S3; Methods. Due to a routing error in the Wave 6 questionnaire that resulted in more than 5% of missing data, variables indicating current smoking status (*n* = 965) and current alcohol consumption (*n* = 1113) were not available for analysis. We also controlled for the number of prior waves of participation (associated with practice effects in cognition) and included 12 country dummies.

In order to control for possible bias resulting from non‐response and selective sampling, all multivariate models were weighted using DBS participation weights (specific for apoE4). These weights were calculated as the product of the calibrated cross‐sectional individual weights to match the population distribution of the predictors and an inverse probability weighting factor called the *non‐apoE4* correction factor. This correction factor was obtained from a logit regression model; the dependent variable was equal to 1 if apoE4 data were collected and the independent variables were age, sex, education, and country, based on information collected in Wave 6. Weighted regressions were implemented with the “Survey” Package in R http://r‐survey.r‐forge.r‐project.org/survey/. To understand the influence of sampling bias, analyses were re‐run unweighted (see Supplementary S5; Results).

### 2.5 Statistical methods

The association between apoE4 detection (1, 0) and global cognitive impairment (1, 0) was tested via weighted multivariable logistic regressions, adjusting for the variables mentioned above (i.e., demographic, lifestyle, health‐related conditions, country, and number of previous waves). Significant positive estimates for the “apoE4 detected = 1” variable would suggest increased odds of global cognitive impairment in the sample.

Since the effects of risk factors and apoE4 on cognition may differ across the life span,^3^ we mean‐centered the age variable and let it interact with the detected apoE4 protein variable (apoE4 detected binding × Age). Age groups were created to visually depict a significant interaction (i.e., adults: 50–64 years, young old: 65–74 years, middle old: 75–84 years, oldest old: 85 years+).

**FIGURE 1:**
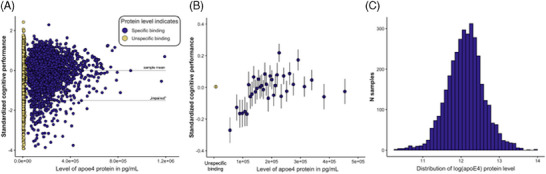
Raw data for apolipoprotein E4 (apoE4) protein levels and cognition. (A) Level of apoE4 protein in pg/mL on the x‐axis, the standardized cognitive composite score on the y‐axis. Higher values on the y‐axis indicate better performance. Horizontal lines highlight the sample mean performance at *y* = 0 and a sample‐specific cutoff of <−1.5 SD, corresponding to a group labeled “global cognitive impairment” in the main text. Participants with protein levels below the cutoff of 30,000 pg/mL are labeled as “Unspecific binding” in yellow. (B) The same data as (A). The cognitive performance within the group of individuals without specific binding is averaged at the furthest left point. Data with specific binding are plotted in bins of *N* = 100. (C) For analyses that use apoE4 protein levels within the “Specific Binding” group, the protein level as a continuous variable was log‐transformed.

The analyses were repeated with the cognitive performance score and individual tests as continuous outcomes in a linear regression analysis. Next, we explored whether, within the subgroup of individuals with detected apoE4, protein level predicted global cognitive impairment. For this purpose, detected apoE4 values were log‐transformed to approximate them to a normal distribution (see Figure 1C) and used as predictors in a weighted logistic regression model. Two separate regressions based on a median split were included to explore non‐linear trends in the data. Finally, we used a weighted linear regression to examine which variables predicted apoE4 levels. This model also controlled for demographic, lifestyle, health‐related conditions, country, and number of previous waves.

Missing values were below 5% for all variables. Multiple imputation for some demographic and health‐related variables and DBS weights was performed using predictive mean matching.^40^ Robustness checks (re‐running unweighted models without imputations) and a list of imputed variables are presented in Supplementary S5 and S6, respectively. Variables with a *p*‐value < 0.05 were considered significant. Statistical analyses were conducted using R (https://www.R‐project.org/).

### 2.6 Data availability

Free access to SHARE data can be granted to registered users through the SHARE Research Data Center (http://www.share‐project.org/data‐access.html). This includes DBS biomarker type‐1 data and fieldwork conditions. Type‐2 biomarkers including apoE4 levels will be included in a future release. The code used to produce the findings and figures of this manuscript is publicly available in https://osf.io/zcuw5/.

## 3 RESULTS

### 3.1 Descriptive data

Table 1 shows participants’ characteristics and descriptive statistics of the sample. Figure 1A shows the concentration of apoE4 protein on the x‐axis and cognitive performance (as a composite Z‐score) on the y‐axis. According to the cutoff of 30,000 pg/mL established in the prior genetic validation of a subsample,^20^ the specific binding of the apoE4 protein was detected in 28.4% of participants, highly consistent with the allelic frequency of the *APOE* ε4 in large European studies.^29^ Figure 1B shows the same data as in 1A, but with cognitive performance averaged across the group of individuals without specific binding of the apoE4 protein and in bins of increasing apoE4 concentration (*N* = 100) for the group in which the specific binding of the apoE4 protein was detected. The horizontal lines in Figure 1A indicate the sample mean cognitive performance at 0 and a cutoff for cognitive impairment at −1.5 SD below the sample mean. 8% of the sample were categorized as “impaired” by the 1.5 SD criterion.

**TABLE 1:** Sample characteristics.

Parameter	Value
Age (years)	67.9 ± 9.2
Sex (*n*, %)	
Male	5099 (40.7%)
Female	7432 (59.3%)
Years of education^a^	11.5 ± 4.3
IADLZA: no problems	11,535 (92%)
IADLZA: 1–3 impaired activities	916 (7.3%)
IADLZA: 4+ impaired activities	80 (0.6%)
Physical activity once a week or more	6451 (51.4%)
Social activities at least every week	4210 (33.6%)
Body mass index (kg/m^2^)	26.8 ± 4.6
Self‐reported medical conditions	
Depressive symptoms^b^	3317 (26.4%)
High cholesterol	3191 (25.4%)
Hypertension	5272 (42%)
1–3 score in CVR	2993 (23.8%)
Current medication	
Hypertensive medication	5687 (45.3%)
Heart medication	1201 (9.5%)
Diabetes medication	1453 (11.5%)
Join pain medication	2080 (16.5%)
Pain medication	1719 (13.7%)
Sleep medication	1027 (8.1%)
Antidepressive medication	886 (7%)
Anti‐inflammatories	388 (3%)
High cholesterol medication	3193 (25.4%)
Country	
Israel	264 (2.1%)
Spain	440 (3.5%)
Greece	312 (2.5%)
Italy	640 (5.1%)
Switzerland	1131 (9.0%)
France	162 (1.3%)
Slovenia	880 (7.0%)
Belgium	1649 (13.2%)
Germany	1385 (11.0%)
Denmark	1937 (15.5%)
Estonia	2341 (18.7%)
Sweden	1391 (11.1%)

*Notes*: *N* = 12,532. Data are presented as means ± standard deviations or number (%).

Abbreviations: CVR, cardiovascular risk index: stroke, diabetes, heart disease; IADLZA, Instrumental Activities of Daily Living score.

^a^ *n* = 12,448 (475 missing cases).

^b^ *n* = 12,448 (83 missing cases).

Next, the raw data shown in Figure 1 were entered in weighted regression analyses to estimate group differences (specific binding of the apoE4 protein detected vs. not detected) and effects of level in the group with detected specific binding, weighted and adjusted for demographic, health and lifestyle variables, and other covariates of no interest.

### 3.2 Associations of apoE4 binding with cognition

**FIGURE 2 alz14224-fig-0002:**
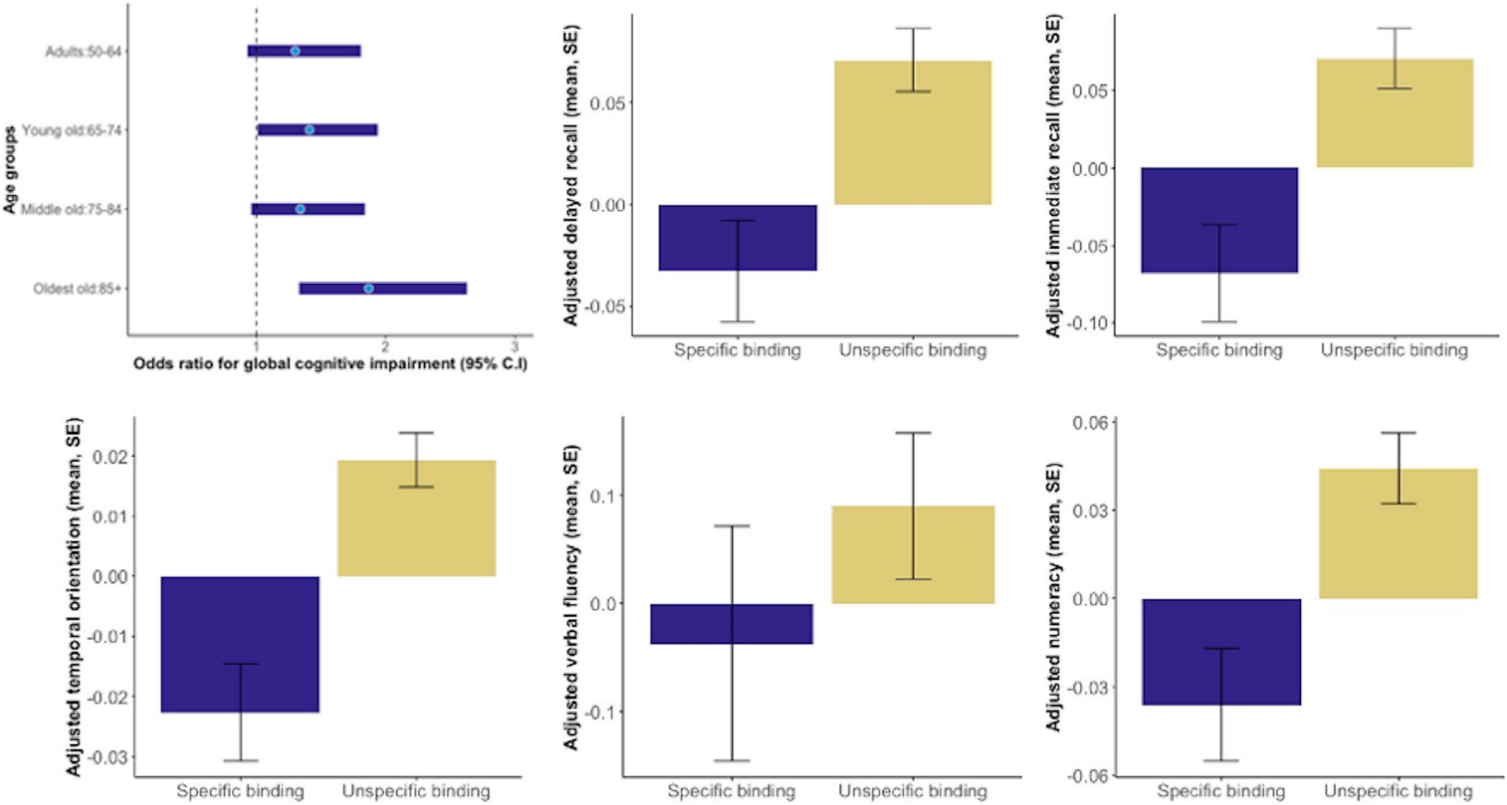
Comparison of cognitive performance by apolipoprotein E4 (apoE4) protein status. Top left: Odds ratios for cognitive impairment (1,0) in relation to apoE4 status (1,0) are pronounced in the oldest group. Other panels: Means and SE for performance on the individual tests, used as continuous outcome variables. Means are based on residuals from the regression adjusted for all covariates. The corresponding tables are in the Supplementary Results S4.

The first weighted binomial model indicated that unspecific versus specific binding of the apoE4 protein (= apoE4 stats 1, 0) is associated with increased odds of global cognitive impairment (odds ratio [OR]: 1.57, 95% confidence interval [CI] = 1.09–2.27, *p* < 0.05, model 1a), particularly in the oldest age group (OR: 1.04, 95% CI = 1.00–1.09, *p* < 0.05, model 1b and Figure 2A), consistent with the literature on *APOE* ε4 genotype and late‐onset AD.^41^ Complete results are reported in Table 2.

**TABLE 2 alz14224-tbl-0002:** Association between detection of apoE4 specific binding in DBS and cognitive status.

Model	Outcome	Predictors	OR	95% CI
1a	Global cognitive impairment	Detected apoE4 specific binding	1.50	1.05–2.13^*^
		Age	1.08	1.06–1.08^*^
		Years of education	0.86	0.82–0.90^*^
		Female sex	1.32	0.91–1.91 n.s
		IADLZA score	1.81	1.33–2.46^*^
		Social activities	0.63	0.42–0.94^*^
		Depressive symptoms	1.74	1.24–2.43^**^
		Physical activity	0.77	0.51–1.15 n.s
		BMI	0.79	0.55–1.13 n.s
		CVR risk score	0.68	0.40–1. 15 n.s.
		Sleep medication	0.38	0.20–0.69^*^
		Other medical conditions (depression, high cholesterol, hypertension)	0.62–1.15	0.28–3.18 n.s.
		Other medications: (cholesterol, hypertension, heart diseases, depression, diabetes, join pain, anti‐inflammatories)	1.09–1.43	0.56–2.30 n.s
1b		Detected ApoE4 specific binding × Age	1.03	1.00–1.07^**^

*Notes*: Demographic, lifestyle, and health‐related predictors are included in each model but only listed once for model 1a. All models controlled for country and number of waves of participation as covariates of no interest. All observations were weighted by the inverse of its probability of being sampled.

Abbreviations: apoE4, apolipoprotein E4; BMI, body mass index; CI, confidence intervals; CVR, cardiovascular risk; IADLZA, Instrumental Activities of Daily Living score; n.s., not significant; OR, odds ratio.

^*^ *p* < 0.05; ^**^ *p* < 0.01; ^***^ *p* < 0.001. *N* = 12,532.

The regression models' variance inflation factors were below 5, indicating no multi‐collinearity between the predictor variables (Supplementary S7; Results).

The (model‐adjusted) mean performance on the individual cognitive tests is shown in Figure 2, separately for the “Specific binding” and “Unspecific binding” groups. For verbal fluency, the group difference was not significant. The corresponding tables for the individual tests and the global cognition composite score as a continuous outcome are reported in Supplementary Results S4.

### 3.3 ole of apoE4 level in individuals with detected specific apoE4 protein binding

Within the group with detected apoE4 protein binding (*n* = 3564), a one‐unit increase in (log) protein level was associated with a decrease in the odds of having global cognitive impairment (OR: 0.47, 95% CI = 0.26–0.85, *p* < 0.05. Table 3). This effect is equivalent to an adjusted OR that suggests a 33.1% decrease in the likelihood of an increase of 1 SD on the protein level (i.e., a protective effect of higher levels). This is, in turn, comparable to the effect of approximately −2 years of age in the same model.[Fig alz14224-fig-0002]

**TABLE 3 alz14224-tbl-0003:** Cognitive status within the group of individuals with detected apoE4 specific binding.

Outcome	Predictors	OR	95% CI
Global cognitive impairment	Log ApoE4 level	0.47	0.26–0.84^*^
	Age	1.14	1.10–1.17^***^
	Years of education	0.86	0.80–0.92^***^
	Female sex	0.97	0.54–1.73 n.s
	IADL	2.11	1.50–2.95^***^
	Social activities	0.42	0.22–0.77^**^
	Depressive symptoms	1.53	0.87–2.69 n.s.
	Physical activity	0.89	0.37–2.13 n.s
	BMI	0.96	0.88–1.03 n.s
	CVR risk score	0.23	0.11–0.48 n.s.
	Sleep medication	0.15	0.05–0.44^***^
	Other medical conditions (depression, high cholesterol, hypertension)	0.36–1.36	0.16–3.70 n.s.
	Other medications: (cholesterol, hypertension, heart diseases, depression, diabetes, join pain, anti‐inflammatories)	0.78–2.28	0.14–5.58 n.s

*Notes*: Model controlled for country and number of waves of participation as covariates of no interest. All observations were weighted by the inverse of its probability of being sampled.

Abbreviations: BMI, body mass index; CI, confidence intervals; CVR, cardiovascular risk; IADL, instrumental activities of daily living; n.s., not significant; OR, odds ratio.

^*^ *p* < 0.05; ^**^ *p* < 0.01; ^***^ *p* < 0.001. *N* = 3564.

Since the plot of the raw data in Figure 1A, B suggests that the protective effect is not linear throughout the group with specific binding, we fitted separate regressions for log(apoE4) versus the cognitive performance Z‐score on each side of a median split on apoE4 level. While the regression without covariates was highly positive for the individuals below the median (*β* = 0.24; *p* < 0.01), after accounting for sample weights and covariates age, sex, education, country, and wave of participation, the effect was reduced to a trend (*β* = 0.13; *p* = 0.08). In contrast, for individuals with apoE4 levels above the median, the association was not significant before or after adding covariates (*ps* > 0.3). The median apoE4 in the detected group was at 183,713 pg/mL (cf. Figure 1B for the raw data).[Table alz14224-tbl-0002]

Taken together with the group comparison reported in Section 3.2, this suggests that having the apoE4 protein, as indicated by specific binding (apoE4 protein level ≥ 30,000 pg/mL), is a risk factor for cognitive impairment. This is in line with literature on the carriage of the *APOE*‐ε4 allele.^2^ However, in those who have the apoE4 protein, high levels of the protein protect the carrier from the risk for low cognitive performance.[Table alz14224-tbl-0003]

The final set of analyses explored predictors of apoE4 levels. The intake of medication against hypertension was associated with lower levels of the apoE4 protein (*β* = −0.11; *p* < 0.05. Complete results in Supplementary S8).

To note, in unweighted multiple regression models, additional variables (age, sex, BMI, diabetes, and high cholesterol) were also significant predictors of apoE4 levels (see Supplementary S5; Table 3; Results). Since sampling weights are used to correct for biases in the cohort composition and provide accurate population estimates, the results from unweighted regressions are not interpreted further.

## 4 DISCUSSION

Animal^11^, ^42^ and human^18^, ^19^, ^22^, ^43^ studies have demonstrated a correspondence between *APOE* ε4 allele and circulating apoE4 levels, that is, only individuals with at least one ε4 allele express apoE4 that is detected by blood assays. Building on this prior knowledge, SHARE Wave 6 employed a multiplex sandwich immunoassay to detect the circulating apoE4 isoform in DBS samples. Our prior work in a subsample of the current study showed that levels higher than 30,000 pg/mL can be labeled as “specific binding detected” and are a highly reliable proxy for carrying at least one *APOE* ε4 allele.^20^ Using this cutoff, and in line with the genetic literature,^2^ the group with detected apoE4 protein‐specific binding had a 57% higher risk of a global cognitive impairment, captured by a binarized composite score based on available cognitive assessment from SHARE. Thus, the first major contribution of the current population study is to demonstrate that the detection of circulating apoE4 above a level of 30,000 pg/mL, assessed from DBS in a population‐based survey, shows associations with cognition, independent of other known risk factors of cognitive decline.

Using this grouping, future research may extend the results of this study and examine the interaction of the apoE4 protein status, obtained with our multiplex assay, with a host of other additional information from the rich SHARE database, including childhood information and longitudinal estimates of lifestyle and health‐related variables. Here, we also identified that the effects on cognition manifested itself most clearly at very old age. These results are comparable to other large genetic population‐based studies, which show that the detrimental effects of the *APOE* ε4 allele are only noted towards the end of the sixth decade of life.^3^, ^44^

In further analyses, utilizing the fact that apoE4 protein levels in those with specific binding varied greatly among the 28.4% of individuals who have it, we found that high apoE4 levels were protective of global cognitive impairment. Inter‐individual differences in apoE4 level were, in turn, modulated by cardiovascular medications. To note, all the models reported in this paper used weighted regressions, thereby controlling, at least in part, for sampling bias that often impacts cognitive aging research.^45^

Previous work linking circulating apoE4 and cognition was conducted mostly in small samples of patients and cognitively impaired individuals and has yielded conflicting results. For instance, some studies reported negative repercussions for cognition with lower circulating levels of apoE4.^11^, ^43^, ^46^, ^47^ Other studies reported no change or diagnostic significance for circulating apoE protein levels and its different isoforms.^22^, ^27^, ^48^

Although carriage of the *APOE* ε4 allele generates a version of circulating apoE with a shorter half‐life, it is plausible that, in the group of participants that express apoE4, higher levels are indicative of higher total apoE protein, suggesting a benefit of high levels of apoE regardless its isoform. In this way, our findings align with the body of research that has identified a general physiological protective function of the apoE protein itself ^10^, ^11^, ^47^, ^49^; higher levels of apoE indicate a potentially more efficient lipid metabolism and better regulation, aggregation, and clearance of Aβ deposition.^10^, ^50^ Dividing the data further by median split, made possibly by our large sample size, suggested the protective effect of high levels of plateaus. This might indicate that there is a limit to a physiological advantage imparted by high levels of apoE or that there are genetic combinations within the apoE4 group (e.g., homozygous) that affect a general linear fit. Only future work that assesses apoE4 level in relation to other isoforms will be able to address the nature of these associations conclusively.

If lifestyle and health factors are associated with higher apoE4 levels, this could explain why some individuals who carry one *APOE* ε4 allele do not develop AD. To this end, we explored what potentially modifiable factors are predictive of apoE4 level.

A significant predictor of lower levels of apoE4 in our study was the self‐reported intake of antihypertensive medication. This could reflect an earlier doctor's diagnosis of poor cardiovascular health that has led to the prescription of medicine. In keeping with a link between cardiovascular health and apoE levels, this may explain why physical interventions seem to boost the effect of protein in the control of hyperlipidemia.^51^, ^52^ However, we did not observe any effect of the measure of physical activity in apoE4 levels, perhaps due to the nature of the self‐reported variable for physical activity in this survey setting. Moreover, SHARE does not have any measure of blood pressure to confirm the medical diagnosis of the participants objectively. Therefore, we are cautious in interpreting these findings further. Future research should examine apoE4 blood levels as an additional outcome of intervention trials that target cardiovascular and metabolic health and examine whether apoE4 levels are amendable.

About the diagnostic value of our cognitive assessment: although this specific battery has not been used in clinical settings, very similar assessments have shown diagnostic value in large longitudinal population studies.^32^, ^33^ Therefore, we are confident that our global cognitive score captured, at least to some extent, the degree of impairment of the respondents. The addition of variables accounting for the number of participation waves controlled for potential effects of learning or repetition. By showing the feasibility of monitoring cognition and apoE4 protein levels in large populations, our study opens new avenues for the understanding of the ecological variables that either contribute to or protect against cognitive decline.

Finally, we stress the use of weighted regressions in this study. The fact that other predictors (age, sex, BMI, high cholesterol), even though biologically plausible, are significant predictors of apoE4 level before but not after weighting, suggests that participation in this kind of studies is likely not random but highly sensitive to selection issues, which may be due to refusal to participate or differential survival up to the moment of study initiation.^45^ For instance, *APOE* ε4 carriers older than 90 years old are a unique group to study resilience factors,^53^ but it is unlikely that this age group is adequately represented in a population‐based study.^54^ Weighted regressions attempt to counteract this sampling bias.

Our study has some limitations. First, SHARE is a population‐based survey that collects medical information, mostly as self‐reports. As such, we acknowledge the lack of specificity of some responses that would allow us to examine in detail how for example, estrogen^55^ or statins against high cholesterol^56^ modulate the circulating levels of apoE4. Second, although SHARE provides a rich set of variables to explain different domains in our models, a more comprehensive cognitive assessment would be beneficial to fully characterize the cognitive status of the participants according to clinical guidelines like mild cognitive impairment (MCI^57^) and multiple domains. Furthermore, longitudinal observations may offer a fuller characterization of the cognitive state of the respondents and should be considered in future studies. Future work in animals is also needed to understand the exact mechanisms by which circulating apoE4 levels and brain apoE4 levels are related and influence cognition across the lifespan.

In conclusion, our analysis from a large population‐based study highlights blood apoE4 levels as a potentially modifiable risk factor for cognitive impairment and could support the development of novel strategies that target individuals who carry the *APOE* ε4 risk gene for AD.

## CONFLICT OF INTEREST STATEMENT

The authors declare no conflicts of interest. Author disclosures are available in the Supporting information.

## CONSENT STATEMENT

Respondents gave informed consent which could be revoked at any time. Wave 6 of SHARE was reviewed and approved by the Ethics Council of the Max Planck Society. In addition, collection and treatment of blood samples had to be approved by country‐specific ethics committees.^31^

## Supporting information

Supporting information

Supporting information
