# Supplementary material for: Circulating apoE4 protein levels from dried blood spots predict cognitive function in a large population‐based survey setting
Source: Alzheimers Dement. 2024 Sep 5;20(11):7613–23. doi: 10.1002/alz.14224 (PMC11567846; doi:10.1002/alz.14224)
Supplement: Supplementary file 1 — Supporting information [file ALZ-20-7613-s001.docx]

# **Supplemental material for**

**Circulating apoE4 protein levels from dried blood spots predict cognitive function in a large population-based survey setting**

Yacila I. Deza-Lougovski^a,b^, Luzia M. Weiss^b^, Hannah M. Horton^b,c^, Aijing Sun^b,c^, Nis Borbye-Lorenzen^d^, Kristin Skogstrand^d^, Solveig Holmgaard^d^, Karen Andersen-Ranberg^e,f^, Vania Panes Lundmark^g,h^, Axel Börsch-Supan^b,c^, Martina Börsch-Supan^c,i*^ & Anna Rieckmann^a,b*^

^a^ Institute of Psychology, University of the Bundeswehr München, 85579 Neubiberg, Germany.

^b^ Max Planck Institute for Social Law and Social Policy, 80799 Munich, Germany.

^c^ Munich Research Institute for the Economics of Aging and SHARE Analyses (MEA), 80804 Munich, Germany.

^d^ Department for Congenital Disorders, Center for Neonatal Screening, Statens Serum Institut, 2300 Copenhagen, Denmark.

^e^ Department of Public Health Epidemiology, Biostatistics and Biodemography, University of Southern Denmark, 5000 Odense, Denmark.

^f^ Department of Clinical Research Geriatric Research Unit, University of Southern Denmark, 5000 Odense, Denmark.

^g^ Department of Integrative Medical Biology, Umeå University, 90187 Umeå, Sweden.

^h^ Umeå Center for Functional Brain Imaging, Umeå University, 90187 Umeå, Sweden.

^i^ Survey of Health, Ageing and Retirement in Europe (SHARE Biomarker Project), Munich, Germany

* Shared senior authorship

## **Supplemental Methods**

## **S1. ApoE4 detection and genetic validation**

A multiplex immunoassay to detect apoE4 protein in SHARE DBS samples was developed. This kind of immunoassays are an ideal method when multiple protein biomarkers are measured in the same sample^1^ with limited material such as DBS. DBS extracts were analyzed with a multiplex immunoassay using preprinted Meso-Scale plates (Meso-Scale Diagnostics (MSD), Maryland, USA) coated with antibodies specific for Apolipoprotein E4 - ApoE4, brain-derived neurotrophic factor - BDNF, Clusterin, Epidermal growth factor - EGF, several interleukins (IL-8, IL-12/23p40, IL-16, IL-18), Monocyte Chemoattractant Protein-1- MCP-1, and Vascular endothelial growth factor - VEGF. From approximately 16,000 DBS assayed samples, binding to the apoE4 antibody could be measured in 12,988 samples (set at 3672 pg/mL for the lower and at 6,250,000 pg/mL for the upper limit) and appeared in two distributions, which were defined as ApoE4-positive and -negative, respectively. From genotyping analyses, we observed that levels higher than 30,000 pg/mL corresponded to carriers of at least one *APOE* ε4. Further information is provided in.^2^

## **S2. Cognitive variables used for the composite score of global cognitive impairment.**

The following available tests available from SHARE Wave 6 were used to create a composite score that indicated global cognitive impairment, based on.^3^

**- Verbal episodic memory:** A word recall test, similar to the Rey’s Auditory Verbal Learning Test (RAVLT^4^) was used to evaluate verbal episodic memory. After an initial presentation of 10 unrelated nouns, respondents were asked to recall as many words as possible within one minute (immediate recall) and again after about five minutes (delayed recall). Correct responses were scored with 1. Scores were computed separately for the immediate and delayed recall tests.

- **Temporal orientation:** Respondents were asked for the current date, month, year, and day of the week. Each correct response was scored with 1, while incorrect responses were scored with 0 (maximum score = 4). The assessment of temporal orientation is comparable to the opening questions of the Mini-Mental State Examination (MMSE), widely used as a screening instrument to evaluate the degree of deterioration of the respondents in clinical settings.^5^

**- Verbal fluency:** Respondents were asked to name as many animals as possible within 60 seconds. The number of correct and non-repeated animal names was counted by the interviewer as the total score of the test. The semantic category “animals” was chosen due to its clear coherence across languages and cultures.^6^

**- Numeracy (subtraction):** In this study, we used the score of the serial subtraction task, which did not have many missing observations. In this task, respondents were asked to perform five serial subtractions beginning with the number 100. One point was giving to each correct answer (maximum score = 5).^8^

# **S3. Interview items and scoring used in the study.**

- **Physical activity:** Participant were asked, how often do they engage in vigorous physical activity, such as sports, heavy housework, or a job that involves physical labour. For the present study, responses were binarized: 1 meant “once a week” or “more than once a week” and 0 meant “one to three times a month” or “hardy ever or never”.
- **Instrumental Activities of Daily Living index - IADLZA:** The version of the ADL index used in SHARE is an adaptation from^7^ and describes the number of limitations with six activities of daily living^8^. A high index means more difficulties with these activities and a lower mobility of the respondent.
- **Social activities:** This question asks for the frequency of engagement in social activities such as voluntary/charity work, attended an educational or training course, gone to a sport/social/other kind of club, or taken part in a political/community-related organization during the past year (almost daily, almost every week, almost every month, or less often).
- **EURO-D depression Scale:** This scale measures clinical depression and contains 12 items tapping into depression, pessimism, suicidality, guilt, sleep quality, interest, irritability, appetite, fatigue, concentration enjoyment, and tearfulness ^9^. In the current study, the score was binarized to indicate the presence (1) or absence (0) of clinically relevant depressive symptomatology.
- **Cardiovascular risk index (CVR):** This score was adapted from^10^ and considers the presence of stroke, myocardial infarction and diabetes. One point is assigned for each disease; the maximum score is 3.
- **Self-declared medical diagnosis:** This question asks whether the respondent received a medical diagnosis and/or is currently under treatment for a list of conditions which included: diabetes, hypertension, high cholesterol, stroke, heart diseases, hip fracture, kidney diseases, ulcer, depression, cataracts, Parkinson, cancer. Only conditions related to cardiovascular health and depression were considered as covariates in our study.
- **Medication intake:** In this question, the respondents were asked whether they take a drug for any medical condition, at least once a week.

# **Supplemental Results**

## **S4. Association of detected ApoE4 protein with individual tests**

| **Delayed recall** | | |
| --- | --- | --- |
| **Predictors** | **β-coefficient** | **95% C.I.** |
| Detected ApoE4 protein | -0.10 | -0.16 – 0.04 * |
| Age | -0.05 | -0.06 – -0.05 * |
| Years of education | 0.08 | 0.08 – 0.09 * |
| Female sex | 0.41 | 0.35 – 0.46 * |
| Instrumental Activities of Daily Living index | -0.39 | -0.44 – -0.33 * |
| Social activities | 0.21 | 0.17 – 0.25 * |
| Physical activity | 0.03 | -0.02 – 0.08 n.s. |
| Depressive symptoms | -0.20 | -0.27 – -0.14 * |
| CVR index | -0.06 | -0.14 – 0.00 n.s. |
| Hypertension | 0.04 | -0.05 – 0.14 n.s. |
| High cholesterol | 0.06 | -0.01 – 0.15 n.s. |
| Body mass index | 0.00 | -0.00 – 0.01 n.s. |
| Affective disorder | -0.16 | -0.28 – -0.03 *** |
| Diabetes meds | -0.00 | -0.11 – 0.10 n.s. |
| Cardiovascular meds | -0.00 | -0.09 – 0.08 n.s. |
| Pain meds | -0.08 | -0.16 – -0.00*** |
| Sleep meds | 0.01 | -0.08 – 0.11 n.s. |
| Joint-pain meds | -0.10 | -0.17 – 0.02 ** |
| Depression meds | 0.03 | -0.08 – 0.15 n.s. |
| Heart meds | -0.06 | -0.16 – 0.03 n.s. |
| Hypertension meds | -0.04 | -0.14 – 0.05 n.s. |
| Anti-inflammatories | -0.01 | -0.17 – 0.13 n.s. |
| Cholesterol meds | -0.00 | -0.09 – 0.08 n.s. |
| Wave of participation | -0.01 | -0.03 – 0.00 n.s. |
| ApoE4 detected x Age | -0.01 | -0.01 – 0.00 *** |

R^2^ = 0.29. C.I. = Confidence intervals. CVR = Cardiovascular risk index. Model controlled for country as a covariate of no interest. *p < 0.001; **p < 0.01; ***p < 0.05; n.s. = not significant.

| **Immediate recall** | | |
| --- | --- | --- |
| **Predictors** | **β-coefficient** | **95% C.I.** |
| Detected ApoE4 protein | -0.15 | -0.39 – -0.03 * |
| Age | -0.07 | -0.08 – -0.06 * |
| Years of education | 0.10 | 0.07 – 0.11 * |
| Female sex | 0.50 | 0.24 – 0.59 * |
| Instrumental Activities of Daily Living index | -0.34 | -0.50 – -0.09 ** |
| Social activities | 0.26 | 0.20 – 0.44 * |
| Physical activity | 0.05 | -0.06 – 0.27 n.s |
| Depressive symptoms | -0.26 | -0.43– -0.07 * |
| CVR index | -0.08 | -0.05 – 0.37 n.s. |
| Hypertension | 0.02 | -0.19 – 0.39 n.s. |
| High cholesterol | 0.15 | -0.21 – 0.16 ** |
| Body mass index | -0.00 | -0.11 – 0.31n.s. |
| Affective disorder | -0.11 | -0.49 – 0.27 n.s. |
| Diabetes meds | -0.05 | -0.19 – 0.07 n.s. |
| Cardiovascular meds | -0.29 | -0.22 – 0.00 *** |
| Pain meds | 0.06 | -0.08 – 0.11 n.s. |
| Sleep meds | 0.11 | -0.09 – 0.15 n.s. |
| Joint-pain meds | -0.12 | -0.22 – -0.04 ** |
| Depression meds | -0.10 | -0.47 – 0.23 n.s. |
| Heart meds | -0.06 | -0.17 – 0.07 n.s. |
| Hypertension meds | 0.15 | -0.13 – 0.11 n.s. |
| Anti-inflammatories | -0.19 | -0.12 – 0.25 n.s. |
| Cholesterol meds | 0.10 | -0.24 – 0.02 *** |
| Wave of participation | -0.04 | -0.07 – -0.02 n.s. |
| ApoE4 detected x Age | -0.01 | -0.02 – -0.01* |

R^2^ = 0.30. C.I. = Confidence intervals. CVR = Cardiovascular risk index. Model controlled for country as a covariate of no interest. *p < 0.001; **p < 0.01; ***p < 0.05; n.s. = not significant.

| **Temporal orientation** | | |
| --- | --- | --- |
| **Predictors** | **β-coefficient** | **95% C.I.** |
| Detected ApoE4 protein | -0.04 | -0.06 – -0.02 * |
| Age | -0.00 | -0.01 – -0.00* |
| Years of education | 0.00 | 0.00 – 0.01 * |
| Female sex | 0.02 | 0.01 – 0.04 * |
| Instrumental Activities of Daily Living index | -0.19 | -0.21 – -0.18 * |
| Social activities | 0.00 | -0.00 – 0.01 n.s. |
| Physical activity | 0.02 | 0.00 – 0.04 ** |
| Depressive symptoms | -0.03 | -0.05 – -0.01** |
| CVR index | -0.02 | -0.04 – 0.00 n.s. |
| Hypertension | 0.03 | 0.01 – 0.06 *** |
| High cholesterol | 0.00 | -0.02 – 0.02 n.s. |
| Body mass index | 0.00 | -0.00 – 0.00 n.s. |
| Affective disorder | -0.03 | -0.07 – 0.00 *** |
| Diabetes meds | 0.04 | 0.01 – 0.07 n.s. |
| Cardiovascular meds | 0.00 | -0.01 – 0.03 n.s. |
| Pain meds | 0.02 | 0.00 – 0.05 *** |
| Sleep meds | 0.03 | 0.00 – 0.06 *** |
| Joint-pain meds | 0.00 | -0.02 – 0.02 n.s. |
| Depression meds | -0.01 | -0.04 – 0.02 n.s. |
| Heart meds | -0.01 | -0.04– 0.01 n.s. |
| Hypertension meds | 0.00 | -0.03 – 0.02 n.s. |
| Anti-inflammatories | 0.00 | -0.04 – 0.05 n.s. |
| Cholesterol meds | 0.00 | -0.01 – 0.03 n.s. |
| Wave of participation | -0.00 | -0.00 – 0.00 n.s. |
| ApoE4 detected x Age | -0.00 | -0.01 – -0.00 n.s. |

R^2^ = 0.10. C.I. = Confidence intervals. CVR = Cardiovascular risk index. Model controlled for country as a covariate of no interest. *p < 0.001; **p < 0.01; ***p < 0.05; n.s. = not significant.

| **Verbal Fluency** | | |
| --- | --- | --- |
| **Predictors** | **β-coefficient** | **95% C.I.** |
| Detected ApoE4 protein | -0.09 | -0.34 – 0.15 |
| Age | -0.19 | -0.21 – -0.18 * |
| Years of education | 0.34 | 0.31 – 0.37 * |
| Female sex | 0.38 | 0.15 – 0.62 ** |
| Instrumental Activities of Daily Living index | -1.34 | -1.56 – -1.12 * |
| Social activities | 1.07 | 0.89 – 1.26 * |
| Physical activity | 0.61 | 0.36 – 0.86 * |
| Depressive symptoms | -0.86 | -1.14 – -0.58 * |
| CVR index | -0.37 | -0.69 – -0.04 *** |
| Hypertension | 0.28 | -0.14 – 0.71 n.s. |
| High cholesterol | 0.04 | -0.32 – 0.41 n.s. |
| Body mass index | -0.00 | -0.03 – 0.02 n.s. |
| Affective disorder | -0.79 | -1.34 – -0.25 ** |
| Diabetes meds | -0.41 | -0.89 – 0.06 n.s. |
| Cardiovascular meds | -0.09 | -0.48 – 0.29 n.s. |
| Pain meds | -0.19 | -0.54 – 0.15 n.s. |
| Sleep meds | -0.15 | -0.60 – 0.29 n.s. |
| Joint-pain meds | -0.50 | -0.82 – -0.17 ** |
| Depression meds | 0.12 | -0.39 – 0.64 n.s. |
| Heart meds | -0.01 | -0.44 – 0.41 n.s. |
| Hypertension meds | -0.36 | -0.79 – 0.07 n.s. |
| Anti-inflammatories | 0.10 | -0.55 – 0.76 n.s. |
| Cholesterol meds | -0.05 | -0.43 – 0.33 n.s. |
| Wave of participation | -0.00 | -0.08 – 0.07 n.s. |
| ApoE4 detected x Age | -0.03 | -0.06 – 0.00 *** |

R^2^ = 0.31. C.I. = Confidence intervals. CVR = Cardiovascular risk index. Model controlled for country as a covariate of no interest. *p < 0.001; **p < 0.01; ***p < 0.05; n.s. = not significant.

| **Numeracy** | | |
| --- | --- | --- |
| **Predictors** | **β-coefficient** | **95% C.I.** |
| Detected ApoE4 protein | -0.06 | -0.10 – -0.01 ** |
| Age | -0.01 | -0.01 – -0.00 * |
| Years of education | 0.05 | 0.04 – 0.05 * |
| Female sex | -0.18 | -0.22 – -0.14 * |
| Instrumental Activities of Daily Living index | -0.27 | -0.31 – -0.23 * |
| Social activities | 0.06 | 0.03 – 0.09 * |
| Physical activity | 0.03 | -0.01 – 0.07 n.s |
| Depressive symptoms | -0.23 | -0.28 – -0.18 * |
| CVR index | 0.05 | -0.11 – 0.00 n.s. |
| Hypertension | 0.02 | -0.05 – 0.09 n.s. |
| High cholesterol | -0.02 | -0.08 – 0.04 n.s. |
| Body mass index | 0.00 | -0.00 – 0.00 n.s. |
| Affective disorder | -0.13 | -0.22 – -0.03 ** |
| Diabetes meds | 0.04 | -0.56 – 0.00 n.s. |
| Cardiovascular meds | -0.01 | -0.04 – 0.12 n.s. |
| Pain meds | -0.05 | -0.11 – 0.00 n.s. |
| Sleep meds | 0.11 | 0.03 – 0.19 ** |
| Joint-pain meds | -0.02 | -0.07 – 0.03 n.s. |
| Depression meds | -0.02 | -0.06 – 0.11 n.s. |
| Heart meds | 0.00 | -0.06 – 0.08 n.s. |
| Hypertension meds | 0.01 | -0.06 – 0.08 n.s. |
| Anti-inflammatories | 0.04 | -0.06 – 0.16 n.s. |
| Cholesterol meds | 0.00 | -0.06 – 0.07 n.s. |
| Wave of participation | 0.00 | -0.01 – 0.01 n.s. |
| ApoE4 detected x Age | -0.00 | -0.01 – -0.00 ** |

R^2^ = 0.19. C.I. = Confidence intervals. CVR = Cardiovascular risk index. Model controlled for country as a covariate of no interest. *p < 0.001; **p < 0.01; ***p < 0.05; n.s. = not significant.

| **Global Cognition Impairment (continuous Z-mean score)** | | |
| --- | --- | --- |
| **Predictors** | **β-coefficient** | **95% C.I.** |
| Detected ApoE4 protein | -0.05 | -0.07 – -0.03 * |
| Age | -0.02 | -0.02 – -0.02 * |
| Years of education | 0.03 | 0.03 – 0.04 * |
| Female sex | 0.08 | 0.06 – -0.10 * |
| Instrumental Activities of Daily Living index | -0.23 | -0.25 – -0.22 * |
| Social activities | 0.08 | 0.07 – 0.10 * |
| Physical activity | 0.04 | 0.02 – 0.06 * |
| Depressive symptoms | -0.12 | -0.14 – -0.09 * |
| CVR index | -0.04 | -0.11 – 0.00 **. |
| Hypertension | 0.03 | -0.05 – 0.09 n.s. |
| High cholesterol | 0.02 | -0.08 – 0.04 n.s. |
| Body mass index | 0.00 | -0.00 – 0.00 n.s. |
| Affective disorder | -0.08 | -0.22 – -0.03 * |
| Diabetes meds | 0.00 | -0.03 – 0.04 n.s. |
| Cardiovascular meds | -0.01 | -0.04 – 0.02 n.s. |
| Pain meds | -0.00 | -0.03 – 0.02 n.s. |
| Sleep meds | 0.03 | -0.00 – 0.07 n.s. |
| Joint-pain meds | -0.03 | -0.06 – -0.01 **. |
| Depression meds | 0.00 | -0.04 – 0.04 n.s. |
| Heart meds | -0.01 | -0.05 – 0.01 n.s. |
| Hypertension meds | -0.01 | -0.05 – 0.02 n.s. |
| Anti-inflammatories | 0.01 | -0.04 – 0.07 n.s. |
| Cholesterol meds | -0.01 | -0.04 – 0.02 n.s. |
| Wave of participation | -0.00 | -0.01 – 0.00 n.s. |
| ApoE4 detected x Age | -0.00 | -0.00 – -0.00 * |

R^2^ = 0.41. C.I. = Confidence intervals. CVR = Cardiovascular risk index. Model controlled for country as a covariate of no interest. *p < 0.001; **p < 0.01; ***p < 0.05; n.s. = not significant.

## **S5. Unweighted imputed and non-imputed models**

| 1. **Global Cognitive Impairment. N = 12,532** | | | | |
| --- | --- | --- | --- | --- |
|  | **Imputed data** | | **Non-imputed data** | |
| **Predictors** | **O.R** | **95% C.I.** | **O.R** | **95% C.I.** |
| Detected ApoE4 protein | 1.53 | 1.29 – 1.81* | 1.56 | 1.29 – 1.04 * |
| Age | 1.07 | 1.06 – 1.08* | 1.07 | 1.06 – 1.08 * |
| Years of education | 0.86 | 0.83 – 0.88 * | 0.86 | 0.84 – 0.88 * |
| Female sex | 1.01 | 0.85 – 1.19 n.s. | 0.88 | 0.74 – 1.06 n.s. |
| Instrumental Activities of Daily Living index | 1.76 | 1.61 – 1.93 * | 1.74 | 1.57 – 1.92 * |
| Social activities | 0.56 | 0.46 – 0.68 * | 0.54 | 0.44 – 0.67 * |
| Physical activity | 0.66 | 0.55 – 0.80 * | 0.68 | 0.56 – 0.83 * |
| Depressive symptoms | 1.58 | 1.33 – 1.88 * | 1.54 | 1.28 – 1.86 * |
| CVR index | 1.06 | 0.89 – 1.27 n.s. | 1.04 | 0.85 – 1.27 n.s. |
| Hypertension | 0.71 | 0.55 – 0.92 *** | 0.69 | 0.52 – 0.92 *** |
| High cholesterol | 0.89 | 0.69 – 1.14 n.s. | 0.90 | 0.68 – 1.17 n.s. |
| Body mass index | 0.97 | 0.95 – 0.98 ** | 0.97 | 0.95 – 0.99 ** |
| Affective disorder | 1.49 | 1.10 – 2.02 ** | 1.62 | 1.16 – 2.24 ** |
| Diabetes meds | 0.87 | 0.66 – 1.15 n.s. | 0.81 | 0.59 – 1.10 n.s. |
| Cardiovascular meds | 0.99 | 0.80 – 1.23 n.s. | 0.95 | 0.74 – 1.20 n.s. |
| Pain meds | 1.11 | 0.90 – 1.37 n.s. | 1.11 | 0.89 – 1.39 n.s. |
| Sleep meds | 0.74 | 0.57 – 0.96 *** | 0.72 | 0.54 – 0.96 *** |
| Joint-pain meds | 0.97 | 0.79 – 1.18 n.s. | 0.98 | 0.79 – 1.21 n.s. |
| Depression meds | 1.01 | 0.73 – 1.38 n.s. | 1.00 | 0.71 – 1.41 n.s. |
| Heart meds | 1.15 | 0.91 – 1.46 n.s. | 1.13 | 0.87 – 1.46 n.s |
| Hypertension meds | 1.11 | 0.85 – 1.44 n.s. | 1.15 | 0.86 – 1.54 n.s |
| Anti-inflammatories | 0.90 | 0.58 – 1.37 n.s. | 0.92 | 0.56 – 1.43 n.s. |
| Cholesterol meds | 1.18 | 0.92 – 1.51 n.s. | 0.90 | 0.68 – 1.17 n.s. |
| Wave of participation | 0.95 | 0.90 – 1.01 n.s. | 0.97 | 0.91 – 1.04 n.s. |
| ApoE4 detected x Age | 1.02 | 1.00 – 1.04 * | 1.02 | 1.00 – 1.04 *** |

O.R = Odd ratio. C.I. = Confidence intervals. CVR = Cardiovascular risk index. Model controlled for country as a covariate of no interest. *p < 0.001; **p < 0.01; ***p < 0.05; n.s. = not significant.

| 1. **Global cognitive impairment in the group with detected apoE4 protein. N = 3564** | | | | |
| --- | --- | --- | --- | --- |
|  | **Imputed data** | | **Non-imputed data** | |
| **Predictors** | **O.R** | **95% C.I.** | **O.R** | **95% C.I.** |
| ApoE4 levels (log) | 0.84 | 0.61 – 1.15 n.s. | 0.84 | 0.60– 1.17 n.s. |
| Age | 1.10 | 1.08 – 1.12 * | 1.10 | 1.07– 1.12 * |
| Years of education | 0.87 | 0.84 – 0.90 * | 0.87 | 0.83 – 0.90 * |
| Female sex | 1.07 | 0.79 – 1.45 n.s. | 0.90 | 0.65 – 1.25 n.s. |
| Instrumental Activities of Daily Living index | 1.51 | 1.27 – 1.80 * | 1.48 | 1.21 – 1.81 * |
| Social activities | 0.61 | 0.44 – -0.82 ** | 0.62 | 0.44 – 0.85 ** |
| Physical activity | 0.65 | 0.47 – 0.90 *** | 0.67 | 0.48 – 0.95 *** |
| Depressive symptoms | 1.66 | 1.20 – 2.30 ** | 1.64 | 1.15 – 2.33 ** |
| CVR index | 1.09 | 0.78 – 1.51 n.s. | 1.04 | 0.72 – 1.49 n.s. |
| Hypertension | 0.80 | 0.50 – 1.28 n.s. | 0.75 | 0.45 – 1.27 n.s. |
| High cholesterol | 0.82 | 0.52 – 1.27 n.s. | 0.86 | 0.53 – 1.39 n.s. |
| Body mass index | 0.95 | 0.92 – 0.99 *** | 0.95 | 0.92 – 0.99 *** |
| Affective disorder | 1.48 | 0.80 – 2.68 n.s. | 1.72 | 0.90 – 3.19 n.s. |
| Diabetes meds | 0.68 | 0.38 – 1.17 n.s. | 0.55 | 0.28 – 1.01 n.s. |
| Cardiovascular meds | 0.95 | 0.63 – 1.42 n.s. | 0.95 | 0.61 – 1.45 n.s. |
| Pain meds | 0.95 | 0.63 – 1.42 n.s. | 1.03 | 0.67 – 1.57 n.s. |
| Sleep meds | 0.64 | 0.37 – 1.05 n.s. | 0.72 | 0.41 – 1.21 n.s. |
| Joint-pain meds | 0.82 | 0.56 – 1.18 n.s. | 0.81 | 0.54 – 1.21 n.s. |
| Depression meds | 1.04 | 0.56 – 1.86 n.s. | 1.09 | 0.56 – 2.04 n.s. |
| Heart meds | 1.14 | 0.72 – 1.79 n.s. | 1.10 | 0.66 – 1.80 n.s. |
| Hypertension meds | 0.90 | 0.55 – 1.44 n.s. | 0.97 | 0.57 – 1.64 n.s. |
| Anti-inflammatories | 0.49 | 0.13 – 1.34 n.s. | 0.56 | 0.15 – 1.56 n.s. |
| Wave of participation | 0.99 | 0.89 – 1.10 n.s. | 1.02 | 0.91 – 1.15 n.s. |
| ApoE4 detected x Age | 1.02 | 0.98 – 1.05 n.s. | 1.01 | 0.98 – 1.05 n.s. |

O.R = Odd ratio. C.I. = Confidence intervals. CVR = Cardiovascular risk index. Model controlled for country as a covariate of no interest. *p < 0.001; **p < 0.01; ***p < 0.05; n.s. = not significant.

| 1. **Predictors of apoE4 (log) protein levels N = 3564** | | | | |
| --- | --- | --- | --- | --- |
|  | **Imputed data** | | **Non-imputed data** | |
| **Predictors** | **β-coefficient** | **95% C.I.** | **β-coefficient** | **95% C.I.** |
| Age | -0.00 | -0.00 – -0.00 ** | -0.00 | -0.00 - -0.00 ** |
| Years of education | 0.00 | -0.00 – 0.00 n.s. | 0.00 | -0.00 – 0.00 n.s. |
| Female sex | 0.08 | 0.05 – 0.11 * | 0.08 | 0.05 – 0.11 * |
| Instrumental Activities of Daily Living index | 0.01 | -0.01 – 0.04 n.s. | 0.01 | -0.01 – 0.04 n.s. |
| Social activities | -0.01 | -0.03 – 0.01 n.s. | -0.00 | -0.03 – 0.01 n.s. |
| Physical activity | -0.00 | -0.03 – 0.03 n.s. | -0.00 | -0.03 – 0.03 n.s. |
| Depressive symptoms | 0.02 | -0.01 – 0.06 n.s. | 0.02 | -0.01 – 0.06 n.s. |
| CVR index | 0.00 | -0.04 – 0.04 n.s. | 0.00 | -0.04 – 0.04 n.s. |
| Hypertension | 0.04 | -0.01 – 0.09 n.s. | 0.04 | -0.01 – 0.10 n.s. |
| High cholesterol | 0.04 | -0.00 – 0.09 *** | 0.04 | -0.00 – 0.09 n.s. |
| Body mass index | 0.01 | 0.00 – 0.01 * | 0.01 | 0.00 – 0.01 * |
| Affective disorder | -0.03 | -0.11 – 0.03 n.s. | -0.03 | -0.11 – 0.10 n.s. |
| Diabetes meds | -0.08 | -0.15 – -0.01 *** | -0.08 | -0.15 - -0.01 *** |
| Cardiovascular meds | -0.03 | -0.09 – 0.01 n.s. | -0.03 | -0.09 – 0.01 n.s. |
| Pain meds | 0.00 | -0.04 – 0.05 n.s. | 0.00 | -0.04 – 0.05 n.s. |
| Sleep meds | 0.00 | -0.05 – 0.06 n.s. | 0.00 | -0.05 – 0.06 n.s. |
| Joint-pain meds | -0.00 | -0.05 – 0.03 n.s. | -0.00 | -0.05 – 0.03 n.s. |
| Depression meds | 0.04 | -0.03 – 0.11 n.s. | 0.04 | -0.03 – 0.11 n.s. |
| Heart meds | -0.03 | -0.09 – 0.02 n.s. | -0.03 | -0.09 – 0.02 n.s. |
| Hypertension meds | -0.05 | -0.11 – 0.00 n.s. | -0.05 | -0.11 – 0.00 n.s. |
| Anti-inflammatories | -0.04 | -0.13 – 0.05 n.s. | -0.04 | -0.13 – 0.05 n.s. |
| Cholesterol meds | -0.10 | -0.15 – -0.04 * | -0.10 | -0.15 – -0.04* |
| External temperature | -0.00 | -0.00 - -0.00 * | -0.00 | -0.00 - -0.00 * |
| Blood spot size (mean) | 0.15 | 0.11 – 0.18 * | 0.15 | 0.10 – 0.18 * |

R^2^ = 0.09.C.I. = Confidence intervals. CVR = Cardiovascular risk index. Model controlled for country as a covariate of no interest. *p < 0.001; **p < 0.01; ***p < 0.05; n.s. = not significant.

## **S6. List of imputed variables**

| **Imputed variable** | **N° imputations (missing cases)** |
| --- | --- |
| Body mass index | 215 |
| Years of education | 475 |
| Physical activity | 2 |
| Instrumental Activities of Daily Living index | 1 |
| Depressive symptoms | 83 |
| Medical conditions (diabetes, hypertension, stroke, cholesterol, heart disease, affective disorders) | 5 |
| Current medication (diabetes, hypertension, high cholesterol, heart disease, anti-inflammatory, sleep, pain, joint pain) | 5 |
| Temporal orientation | 1 |
| Verbal fluency | 2 |
| Mean blood spot size | 3 |
| Survey weights | 173 |

## **S7. Variance inflation factors in regression models**

To detect potential multicollinearity in our regression models, we calculated the variance inflation factor (VIF), which is a measure of the correlation and strength of correlation between the predictors in the model. It is calculated with the following formula:

$${VIF}_{j}=\frac{1}{1-R_{j}^{2}}$$

where *R^2^_j_* is the *R^2^*-value obtained by regressing the *j^th^* predictor on the remaining predictors. A value of 1 indicates no correlation, values between 1 and 5 indicate moderate correlation and values greater than 5 indicates potentially severe correlation between a given predictor variable and other predictor variables in the model^11^. The VIFs between all the predictors used in our analyses are detailed below:

| **Predictors** | **Variance inflation factor** |
| --- | --- |
| ApoE4 detected | 1.03 |
| Age | 1.29 |
| Years of education | 1.30 |
| Sex | 1.09 |
| Instrumental abilities | 1.20 |
| Social activities | 1.10 |
| Physical activity | 1.15 |
| Depressive symptoms | 1.22 |
| Cardiovascular risk index | 2.29 |
| Hypertension | 2.84 |
| High cholesterol | 2.30 |
| Body mass index | 1.16 |
| Diabetes meds | 1.76 |
| Cardiovascular meds | 1.27 |
| Pain meds | 1.16 |
| Sleep meds | 1.19 |
| Joint-pain meds | 1.16 |
| Depression meds | 1.43 |
| Heart meds | 1.24 |
| Hypertension meds | 2.85 |
| Anti-inflammatories | 1.05 |
| N° wave of participation | 1.55 |

**S8. Predictors of apoE4 protein levels (weighted model)**

| **Outcome and R^2^** | **Predictors** | **β-coefficient** | **95% C.I.** |
| --- | --- | --- | --- |
| ApoE4 (log)  R^2^ = 0.05 | Age | -0.00 | -0.00 – 0.00 n.s. |
|  | Years of education | 0.00 | -0.00 – 0.01 n.s. |
|  | Female sex | 0.01 | -0.05 – 0.09 n.s. |
|  | Instrumental Activities of Daily Living index | 0.01 | -0.04 – 0.07 n.s. |
|  | Physical activity | -0.06 | -0.15 – 0.02 n.s. |
|  | Social activities | 0.04 | -0.01 – 0.10 n.s. |
|  | Depressive symptoms | 0.03 | -0.06 – 0.14 n.s. |
|  | Body mass index | 0.00 | -0.00 – 0.01 n.s. |
|  | CVR index | -0.04 | -0.16 – 0.05 n.s. |
|  | High cholesterol | 0.02 | -0.07 – 0.13 n.s. |
|  | Hypertension | 0.04 | -0.06 – 0.19 n.s |
|  | Affective disorder | 0.04 | -0.06 – 0.19 n.s. |
|  | Hypertension meds | -0.11 | -0.23 – -0.00 * |
|  | Cardiovascular meds | -0.07 | -0.19 – 0.04 n.s. |
|  | Heart disease meds | 0.01 | -0.12 – 0.14 n.s. |
|  | Diabetes meds | -0.10 | -0.24 – 0.04 n.s. |
|  | Joint-pain meds | -0.03 | -0.12 – 0.05 n.s. |
|  | Pain meds | -0.01 | -0.13 – 0.10 n.s. |
|  | Sleep meds | 0.06 | -0.05 – 0.18 n.s. |
|  | Depression meds | 0.09 | -0.02 – 0.22 n.s. |
|  | Anti-inflammatories | -0.11 | -0.34 – 0.11 n.s. |
|  | External temperature | -0.01 | -0.01 – 0.25 *** |
|  | Blood spot size (mean) | 0.12 | 0.00 – 0.25 * |

C.I. = Confidence intervals. CVR = Cardiovascular risk index. Model controlled for country as a covariate of no interest. Probability weights were included in the regression. **p <*0.001; ***p <*0.01; ****p <*0.05; n.s. = not significant.

**References**

1. Skogstrand K. Multiplex assays of inflammatory markers, a description of methods and discussion of precautions – Our experience through the last ten years. *Methods*. 2012;56(2):204-212. doi:10.1016/j.ymeth.2011.09.025

2. Borbye-Lorenzen N, Deza-Lougovski YI, Holmgaard S, et al. Assessment of circulating apoE4 levels from dried blood spot samples in a large survey setting. *Alzheimer’s & Dementia: Diagnosis, Assessment & Disease Monitoring*. 2024;16(1):e12555. doi:10.1002/dad2.12555

3. Ahmadi-Abhari S, Guzman-Castillo M, Bandosz P, et al. Temporal trend in dementia incidence since 2002 and projections for prevalence in England and Wales to 2040: modelling study. *BMJ*. Published online July 5, 2017:j2856. doi:10.1136/bmj.j2856

4. Harris SJ, Dowson JH. Recall of a 10-Word List in the Assessment of Dementia in the Elderly. *The British Journal of Psychiatry*. 1982;141(5):524-527. doi:10.1192/bjp.141.5.524

5. Woodford HJ, George J. Cognitive assessment in the elderly: a review of clinical methods. *QJM: An International Journal of Medicine*. 2007;100(8):469-484. doi:10.1093/qjmed/hcm051

6. Ardila A, Ostrosky-Solís F, Bernal B. Cognitive testing toward the future: The example of Semantic Verbal Fluency (ANIMALS). *International Journal of Psychology*. 2006;41(5):324-332. doi:10.1080/00207590500345542

7. Katz S, Ford AB, Moskowitz RW, Jackson BA, Jaffe MW. Studies of Illness in the Aged: The Index of ADL: A Standardized Measure of Biological and Psychosocial Function. *JAMA*. 1963;185(12):914-919. doi:10.1001/jama.1963.03060120024016

8. Banks J, J U, Blundell R, Marmot M, Lessof C, Nazroo J. Health, wealth and lifestyles of the older population in England: The 2002 English Longitudinal Study of Ageing. Published online 2004. Accessed May 17, 2023. https://research.manchester.ac.uk/en/publications/health-wealth-and-lifestyles-of-the-older-population-in-england-t-2

9. Prince MJ, Reischies F, Beekman ATF, et al. Development of the EURO–D scale – a European Union initiative to compare symptoms of depression in 14 European centres. *The British Journal of Psychiatry*. 1999;174(4):330-338. doi:10.1192/bjp.174.4.330

10. Kaffashian S, Dugravot A, Elbaz A, et al. Predicting cognitive decline: a dementia risk score vs. the Framingham vascular risk scores. *Neurology*. 2013;80(14):1300-1306. doi:10.1212/WNL.0b013e31828ab370

11. O’brien RM. A Caution Regarding Rules of Thumb for Variance Inflation Factors. *Qual Quant*. 2007;41(5):673-690. doi:10.1007/s11135-006-9018-6
